# iKanEat: protocol for a randomized controlled trial of megestrol as a component of a pediatric tube weaning protocol

**DOI:** 10.1186/s13063-021-05131-w

**Published:** 2021-02-27

**Authors:** Sarah Edwards, Paul E. Hyman, Hayat Mousa, Amanda Bruce, Jose Cocjin, Kelsey Dean, Kandace Fleming, Rebecca Swinburne Romine, Ann M. Davis

**Affiliations:** 1 Pediatric Gastroenterology, Children’s Mercy Kansas City, Kansas City, MO USA; 2 Pediatric Gastroenterology, New Orleans Children’s Hospital, New Orleans, LA USA; 3 Pediatric Gastroenterology, Hepatology and Nutrition, University of California San Diego/Rady Children’s Hospital, San Diego, CA USA; 4 Department of Pediatrics, University of Kansas Medical Center, 3901 Rainbow Boulevard, MS 4004, Kansas City, KS 66160 USA; 5 Center for Children’s Healthy Lifestyles & Nutrition, Kansas City, MO USA; 6 Life Span Institute, University of Kansas, Lawrence, KS USA

**Keywords:** Tube feeding, Tube weaning, Megestrol, Feeding problems, Randomized controlled trial

## Abstract

**Background:**

Although tube feeding routinely saves the lives of children who do not eat by mouth, chronic tube feeding can be a burden to patients, caregivers, and families. Very few randomized trials exist regarding the best methods for weaning children from their feeding tubes.

**Methods:**

The current paper describes a randomized controlled trial of an empirically supported outpatient treatment protocol for moving children from tube to oral eating called iKanEat. Specifically, we describe the methods of randomized double-blind, placebo-controlled trial which includes a 4-week course of megestrol, the only medication used in the iKanEat protocol, to determine whether the addition of megestrol results in improved child outcomes. The primary and secondary aims are to assess the safety and efficacy of megestrol as part of the iKanEat protocol. The third aim is to provide critical information about the impact of the transition from tube to oral feeding on parent stress and parent and child quality of life.

**Discussion:**

This trial will provide data regarding whether megestrol is a safe and effective component of the iKanEat tube weaning protocol, as well as important data on how the tube weaning process impacts parent stress and parent and child quality of life.

**Trial registration:**

ClinicalTrials.gov NCT#03815019. Registered on January 17, 2019

## Background

Gastrostomy (G-) feeding tubes are placed in infants and children who refuse to eat or have inadequate weight gain. Common medical causes of inadequate weight gain include neurological disease, congenital heart defects, chronic pulmonary disease, renal failure, genetic disorders, anatomic abnormalities, behavioral disorders, and oropharyngeal dysphagia [1, 2]. Feeding tubes allow patients to obtain sufficient nutrition [3]. Although the feeding tube provides a way to ensure adequate nutrition, adverse outcomes can include infection of the G-tube insertion site, granulation tissue, and leakage of gastric contents [4]. Some data indicate stress levels of caregivers can increase after G-tube placement [5, 6]. Specifically, parents of tube-fed children encounter multiple psychosocial stressors regarding tube feeding [7], which include concerns about their child’s survival due to their underlying medical issues, feelings of “failure” due to their inability to feed their child orally [8], increased feelings of stress around the tube feeding [9], and decreased support from others due to the tube feeding [10, 11]. It has been demonstrated that initiating the transition from tube to oral feeding as early as possible improves the likelihood of successful tube weaning [3].

Existing treatment options for transitioning from tube to oral feeding include outpatient, inpatient, and day treatment. The most widely available treatment option is outpatient treatment by a multidisciplinary team [12]. These teams include professionals from multiple specialties, including medical (pediatric gastroenterologist or pediatrician), psychology, speech, occupational therapy, and nutrition [13]. Outpatient treatment is typically covered by health insurance and can be tailored to meet the needs of the patient and family. Typically, the patient and family see the providers regularly (i.e., monthly, quarterly) to receive recommendations that are then implemented in the home. This treatment option is effective for some patients, but other children require a more intensive program for the tube weaning process.

Our team developed a novel interdisciplinary outpatient protocol for transitioning children from tube to oral feeding [14, 15] called “iKanEat.” Previous data suggest iKanEat is effective for transitioning tube-fed children to eating by mouth [16] and results in statistically significant and clinically meaningful increases in oral eating. iKanEat was originally composed of several key components, including two medications—amitriptyline and megestrol. However, our most recent work (NIH HD066629) [16] demonstrated that amitriptyline is not a necessary component of the protocol, as all children who completed the protocol consumed 100% of their calories orally at post-treatment regardless of receiving amitriptyline or placebo. Amitriptyline was subsequently removed from the iKanEat protocol. The current protocol is a randomized controlled trial of the second medication (megestrol) compared to placebo, to assess whether the addition of a 4-week course of megestrol improves child outcomes within the iKanEat protocol. Because corticosteroids can have significant side effects, it is critical to determine if the benefits of megestrol as part of iKanEat outweigh the risks of the medication. It is imperative that we determine the efficacy and safety of the protocol including 4 weeks of megestrol before we move toward broad dissemination.

Megestrol is a steroid and progestational drug approved by the FDA for treating anorexia or weight loss in adults with acquired immunodeficiency syndrome. The precise mechanism of action that leads to increased appetite and weight gain is unknown, but is likely related to megestrol’s glucocorticoid effect [10]. Side effects of megestrol may include suppression of the adrenal glands and new-onset diabetes mellitus [10]. These complications are minimized by limiting the duration of treatment to 6 weeks [10]. Other side effects may include heart failure, nausea and vomiting, edema, dyspnea, hyperglycemia, alopecia, hypertension, carpal tunnel syndrome, mood changes, malaise, asthenia, lethargy, sweating, and rash [10].

Megestrol’s safety and efficacy have not been established in children. However, there are several pediatric trials with megestrol for anorexia or malnutrition [11, 17–20]. Two pediatric trials have focused on megestrol to increase appetite in cancer patients [17, 18]. Cuvelier and colleagues showed clinically significant effects of megestrol on weight gain (+ 19.7% versus placebo − 1.3%). Only two of the 13 megestrol patients developed severe adrenal insufficiency [17]. The other study employed megestrol as a second-line treatment if the child did not respond to cyproheptadine (Periactin) [18]. Five of the seven patients responded positively to megestrol, and only one showed adrenal suppression [18]. A retrospective study by Orme and colleagues again demonstrated the effectiveness of megestrol in appetite, caloric intake, and weight gain, but the majority of the patients exhibited some effects of adrenal suppression; one exhibited clinically significant adrenal suppression [19]. These adrenal changes are reversible once the medication is discontinued, as demonstrated by a study with children with cystic fibrosis [20]. In this randomized control trial of patients with cystic fibrosis, patients in the treatment group reached 100% of their ideal body weight within 3 months of initiating therapy and also improved their pulmonary function [20]. While the majority showed signs of adrenal suppression, it was reversible following cessation of treatment [20]. In a megestrol trial for children with cystic fibrosis, the youngest child treated was 6 months of age and treated with 10 mg megestrol/day or placebo for 12 weeks [11].

The primary aim of the current study is to conduct a randomized double-blind, placebo-controlled trial of a 4-week course of megestrol as a component of the iKanEat protocol. The primary aim is to assess whether the addition of megestrol improves child outcomes. The second aim is to assess the safety of megestrol as part of the iKanEat protocol. Our previous work (as well as work by others) suggests that a 6-week course of megestrol can lead to adrenal insufficiency in some children [16–19], so as part of the current protocol, we will assess the safety of a 4-week course of this drug. Finally, the third aim of the study is to examine the effect of the tube to oral transition on parent stress and parent and child quality of life.

## Methods

### Specific aims and hypotheses

#### Aim 1

To assess the efficacy of megestrol as part of the 24-week iKanEat protocol.

#### Hypothesis 1

Children randomized to the megestrol group will be more successful in making the transition to oral feeding (defined as obtaining at least 90% of calories orally) than children randomized to the placebo group.

#### Aim 2

To assess the safety of 4 weeks of megestrol as part of the 24-week iKanEat protocol.

#### Hypothesis 2A

Children randomized to the megestrol group will not differ from control children in morning cortisol classification level (low, average, high) and will remain within the normal range at all time points. Analyses 2B: Exploratory analysis will determine which, if any, covariates (gender, age, diagnoses at week 0, and diagnoses at birth) are related to abnormal morning cortisol levels.

#### Aim 3

To examine the effect of the transition from tube to oral feeding on parent stress and parent and child quality of life.

#### Hypothesis 3A

The transition to oral feeding will temporarily increase parent stress at week 14 at the cessation of tube feeding; for the children who successfully transition, we hypothesize a return to baseline by week 24.

#### Hypothesis 3B

The successful transition to oral feeding will increase parent/child quality of life at 24 weeks compared to week 0.

### Participant selection and withdrawal

Participants will be identified through multidisciplinary feeding teams at 10 participating sites throughout the USA. Parents of patients who meet the criteria will be approached during feeding team visits or contacted by feeding team staff. Methods of recruitment could include approaching patients during routinely scheduled patient visits, sending letters to patients identified through an electronic medical record or other site-specific patient registry, emailing patients through the electronic medical record platform, or other recruitment methods as allowed by the Institutional Review Board at each site.

Seventy-two children (at least 60 completers) ages 0 year 9 months 0 day through 8 years 31 days will be recruited (see the “Sample size determination” section) with a maximum of one child per family. To ensure an equal balance of male and female across the two groups, the statistical team will evaluate the percentage of male in each group halfway through subject recruitment. If there is not an equal proportion of male/female in each group, the statisticians will implement a change in the allocation tables of the randomization plan to correct this. For inclusion/exclusion criteria, see Table 1, all of which are consistent with the prior iKanEat study (NIH HD066629). Children must receive over 80% of their total daily calorie needs from a tube to be classified as tube dependent. The lower age limit matches our recently completed feeding study. For the current study, we set an upper limit of 8 years 31 days due to limitations of our measures which are only normed through age 6 (meaning the child has to be 6 years or younger at the final assessment point), and to improve the developmental homogeneity of our sample. However, during implementation, the upper age limit was increased to 9.0 years. The child must have a history of feeding problems as identified by a diagnosis from a multidisciplinary feeding team and must have permission from the physician on the team to ensure that they are medically stable enough to participate in a weaning study. Finally, they will be required to have oral motor skills sufficient to promote positive oral eating, including oral skill development at or near the child’s developmental age (as assessed by an Occupational Therapist or Speech Language Pathologist at their site). The child will be excluded if they are receiving oral or inhaled steroids as these can affect adrenal insufficiency. Families will be excluded if the parent has a known developmental delay or cognitive impairment that may make participation in the study difficult (children with these issues will not be excluded). Families will also be excluded if the child is already *currently* receiving *intensive* (defined as more than one session per month) behavioral feeding therapy (previous feeding therapy is not an exclusion criterion). Parents are required to be English speaking because many of our measures are not validated in Spanish or other languages. Children and families will continue all ongoing medical care throughout the study.

**Table 1:** Inclusion/exclusion criteria

Inclusion criteria	Exclusion criteria
Child has a G or G/J tube	Parents have known significant developmental delay or cognitive impairment
Child receives > 80% kcal from the tube	
Child ages 0 year 9 months 0 day through 8 years 31 days	Child is receiving intensive behavioral feeding therapy (> 1 session per month) at the time of consent. Oral motor therapy is not an exclusion.
Physician permission to participate	Parents primary language other than English
Child has sufficient oral motor and behavioral skills for oral eating (see Table 4)	
Child has > 3-month history of feeding problem	Child receiving inhaled or oral steroids
Child has behavioral skills necessary for mealtime (see Table 4)	

A participant will be withdrawn from the study if more than 0.5 kg of weight is lost consistently per week over three consecutive weeks, or if 10% of total body weight is lost. Children will also be removed if they have any serious adverse event, such as significant negative changes in mood, sleep, or food refusal. Children who are removed from the study will be treated clinically.

If a participant drops from the study, they will be asked to continue on with their assessments (at least height and weight and diet recall, at the very minimum). This will be done so we can have outcome on our primary endpoint even for subjects who drop out from treatment. Unless a subject/parent actively refuses, we will try to contact them for these assessments via phone at least 5 times to schedule, and if this fails, we will attempt to reach them via certified letter at their last known address.

### Study procedures

All study procedures will be approved by the Institutional Review Board at the primary site (the University of Kansas Medical Center) and are listed at ClinicalTrials.gov (NCT#03815019) as outlined in Table 2 procedure timeline. An outline of study activities by year is presented in Table 3.

**Table 2:** Procedure timeline

Procedures	Pre *(−14 to − 1 days)*	Week																								
		0	1	2	3	4	5	6	7	8	9	10	11	12	13	14	15	16	17	18	19	20	21	22	23	24
**Patient feeding/drug schedule**																										
Tube feeding via G or G/J			X	X	X	X	X	X	X	X	X															
Tube taper												X	X													
Megestrol or placebo												X	X	X	X											
**Completed at each site**																										
Eligibility	X																									
Informed consent	X																									
Demographics (REDCap)	X																									
Quality of life measures: PIP, ITQOL, PQOL, PedsQL, CHQ (REDCap)	X											X				X										X
Clinic visit		X										X				X										X
Vitals (ht, wt, bp, temp, pulse)		X										X				X										X
Blood draw, morning cortisol												X				X										
Subject payment ($100)		X										X				X										X
Dispense study drug												X														
**Completed by Kansas Team**																										
3-day diet recall	X											X				X										X
Randomization		X																								
Tele-visit (30 min)			X		X		X		X		X		X		X		X		X		X		X		X

**Table 3:**
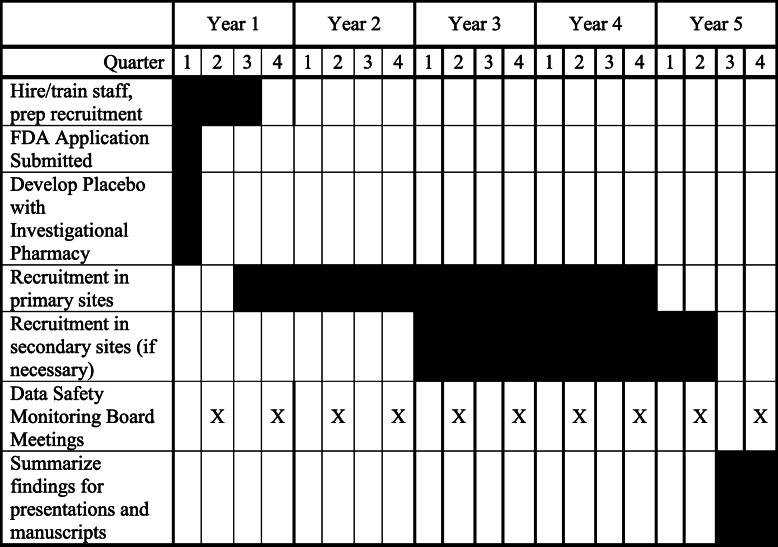
Timeline of study-related activities by year

### Screening visit

Families who meet the criteria for participation (Table 1) will complete the consent process (with the site PI or site research coordinator) and all baseline measures (see the “Measures” section). Following consent and the completion of all baseline measures, the statistical team will conduct blinded random assignment using computer-generated randomization codes and this information will be communicated to the investigational pharmacy only—all other staff will remain blind to group assignment throughout the study, making this a double-blind, placebo-controlled trial.

### Megestrol

The proposed study will use megestrol 6 mg/kg/day in two equal doses for 4 weeks [10, 11, 17]. Megestrol will be dosed based on weight at the week 10 visit. The megestrol will be dosed at full dose weeks 10–11, at 66% dose week 12, at 33% dose week 13, and fully tapered at the end of week 13. The lead investigational pharmacy will coordinate with each site their purchase of the megestrol solution from the single manufacturer. All sites will use the exact formulation, containers, and dosing instructions as specified by the lead investigational pharmacy.

Participants will be given instructions on when to take the medication and what to do if they miss a dose. If it has been < 6 h since missing the dose, they should take the dose as soon as they remember. If it has been ≥ 6 h since missing the dose, they should skip the dose. Vomited doses should be re-dosed if within 30 min of original dosing. Participants will be asked to document any skipped doses and report those to the study team at their next visit.

### Placebo

The investigational pharmacy developed a placebo that is matched to the megestrol suspension in terms of taste, appearance, viscosity, and storage properties. The placebo will have no active ingredients and will be provided in the same containers, with the same labels and instructions for dosing as the active medication.

### Randomization

Patients will be randomized within each site at a 1:1 ratio into the treatment and placebo groups. The statistical team will generate ten sets of randomization codes and send one set of codes to the pharmacist of each site. All the other study personnel will be blinded to the result of randomization.

The pharmacy at each site will prepare megestrol or placebo for each subject as dictated by the random codes prepared by the study statistician. The compound formula for each site will be identical, and the taste, look, and smell of the placebo and megestrol will be matched.

### iKanEat intervention

The 24-week iKanEat intervention is composed of 4 clinic visits and a series of 12 remote tele-visits (see Table 2: procedure timeline). Serious adverse events (SAEs) will be assessed at every point of contact. Clinic visits will occur at each site in their clinical space. Each visit will involve obtaining weight and height (in triplicate, in light clothing), vital signs, and then speaking with a healthcare practitioner regarding overall health. The child will also receive a complete physical exam, similar to a routine pediatric visit, including blood pressure and temperature, and assessed for side effects of the iKanEat protocol. Patients will also be sent to the lab for procurement of blood serum to test for adrenal insufficiency at appropriate time points. At the initial clinic visit, a medical history will also be obtained. Clinic visits will occur at weeks 0, 10, 14, and 24. The megestrol will be dosed at full dose weeks 10–11, at 66% dose week 12, at 33% dose week 13, and fully tapered at the end of week 13. Five days after the week 10 clinic visit, parents will begin to taper G or G/J tube feedings by 10% each day until they are stopped all together. The tube weaning schedule is outlined by the site PI, provided to the family, and documented in the RedCap database.

Tele-visits (audio and video enabled) will begin by building rapport and asking for a summary of all relevant information since the last point of contact, including parent perception of changes in weight, feeding habits, progress, stress of parent/child, and illness. Children will be assessed for side effects of the iKanEat protocol at every tele-visit per parent report. Visits will be scheduled around the child’s mealtime so that a feeding can be observed during the session. The majority of the time left in the 30-min session will be spent dealing with parent concerns, which our previous project indicates may include questions about measures, questions about implementation of the iKanEat protocol, and ensuring that children/families adhere to the oral motor and behavioral guidelines for feeding (see Table 4). Specifically, we will assess for the presence of daily mealtimes (at least 3–5 times per day), limited grazing between planned meals/snacks, consistent mealtime location, appropriate meal length (approx. 20 min), and limited distractions during mealtime (refrain from the use of TV, iPad, toys, etc.). Families will also be encouraged to engage in family mealtime together, to prohibit force feeding across all feeders, and to focus on positive parent behavior, positive meal demeanor, and appropriate food presentation (as defined in Table 4). Motivational interviewing and cognitive-behavioral parent training techniques will be used to improve parent performance on these skill areas. These skills will rely heavily on the detailed training manuals available from The Incredible Years, and our therapists will specifically use the materials from the book *Collaborating with Parents to Reduce Children’s Behavior Problems: A book for Therapists Using the Incredible Years Programs*. The structure and content of these tele-visits is based directly on those used in our prior work (NIH HD066629).

**Table 4:** Definitions of oral motor, sensory, and behavioral skills

**Oral motor skills**	
Age-appropriate strength and coordination of oral cavity	Adequate range of motion, strength and coordinated movement of the lips, tongue, and jaw
Head/neck/trunk support	Strength and control of the head, neck, and trunk to provide midline stability of the body
**Sensory issues**	
Sensory processing	Exposure to overcome sensory processing issues that interfere with daily life activities, specifically eating/feeding
**Behavioral skills**	
Regular meals	Coming to the meal setting at least 2–3 times per day, willingly
Limited grazing	Child does not simply graze throughout the day, but participates in structured family mealtimes
Same location	Daily meals take place in the same location
Meal length	Meal length falls between 10 and 20 min
Meal distractions	There are few distractions during mealtime (i.e., TV) that occur on a routine basis
Family mealtime	The child and family eat meals together on a regular basis
Structured start and end	The parent dictates the start and end of the meal with a simple command such as “It’s time to eat” or “You may get down now”
Parent behavior during meals	Parent behavior during meals is appropriate with limited coaxing and no yelling or threatening
Force feeding	There is never any forcing of food or other objects into the child’s mouth
Meal demeanor	Child is neutral or positive in response to mealtime without crying or constantly turning the head away from the spoon
Good food presentation	Appropriate amount/variety of foods are presented in a calm, relaxed manner; feeders announce each bite

#### Tele-technology

Parents will be allowed to choose if they wish to receive their tele-visits over phone (as we did in our previous work) or via interactive televideo. For patients who choose to do the visits over phone, we will provide a toll-free number with a fully secure connection. For patients who choose to use the tele-visit option, we will use the *Zoom Mobile Meeting Platform* which provides a secure videoconference bridge. These point-to-point connections are secure, meaning there is no concern about the release of protected health information. The Zoom platform also allows for the connection of diverse types of systems, including mobile devices, desktop computers, and established telehealth equipment in many clinical settings (important for eventual dissemination, if appropriate). The Zoom m500 app or a similar secure technology will be utilized to connect participants to the study team for all procedures. No matter whether patients chose to receive their tele-visits over phone or interactive televideo, they will be using their own existing device, such as a home phone, cell phone, desktop computer, or tablet device.

#### Interventionists

Interventionists for the clinic visits will be pediatric gastroenterologists or pediatricians who specialize in feeding, accompanied by study team staff at each site. These physicians are experts in feeding and serve as the physicians and/or Medical Directors of the feeding programs at their sites. Interventionists for the tele-visits will be behavioral experts at the primary site (KUMC) and are trained multidisciplinary feeding team members with telehealth experience, including doctoral-level psychologists or their trainees. All behavioral interventionists are trained in motivational interviewing and in cognitive-behavioral parent training and are familiar with The Incredible Years program that will serve as a basis for the behavioral parent training program used here.

#### Fidelity

Treatment fidelity for tele-visits will be measured by having a Graduate Research Assistant code a randomly selected sample of 10% of all intervention sessions (via recording). They will code for adherence to the visit checklist, which will follow the procedure timeline. The tapes will be selected and coded via a random numbers table. Tapes are easily captured for tele-visits via digital recording for phone and via existing technology for interactive televideo apps.

#### Multi-site study

The current study is proposed at several sites in order to recruit the number of children who meet the specific inclusion/exclusion criteria. Conducting a multi-site study can present hurdles. However, many of the investigators have worked together previously on feeding studies (HD068221) and publications. To enhance protocol adherence across sites, the PI will travel to each site annually, and the team will meet every year at the NASPGHAN meeting. Also, a weekly mandatory 1-h phone conference will occur across all active sites to discuss protocol implementation issues and patient safety. Sites will be given written protocols during orientation that define protocol deviations, unanticipated problems, and adverse events, and the method to report those to the lead site. Sites that have agreed to participate include the University of Kansas Medical Center, Children’s Mercy Hospital, University of California San Diego/Rady Children’s Hospital, Arnold Palmer Hospital for Children, Children’s Hospital of Philadelphia, Vanderbilt University Medical Center, and Boston Children’s Hospital. Additional sites available for recruitment, if the current sites fall behind in their recruitment goals, include the sites of our Data Safety Monitoring Board Members, and sites that are members of the newly formed National Feeding Consortium (of which the PI and several site PIs are members). Sites will all have an existing multidisciplinary feeding team that includes at least a pediatric healthcare provider (MD, DO, ARNP), Psychologist, Dietitian, and Occupational Therapist/Speech Pathologist. The University of Kansas Medical Center will be the lead and coordinating site. Research data will be entered by the sites into a REDCap data capture system managed by the KUMC study team. Each individual site PI and research coordinator will be responsible for recruitment at their site, as well as taking consent.

#### Measures

Data collection will occur as specified in Table 5. As in our previous research, for two parent families, questionnaires will be completed by the primary parent associated with feedings.

**Table 5:** Measures timeline

	Week			
	0	10	14	24
24-h food recall	X	X	X	X
Morning cortisol level		X	X	
The Pediatric Inventory for Parents	X	X	X	X
Infant Toddler Quality of Life	X	X	X	X
Child Health Questionnaire Parent Form	X	X	X	X
Family Impact Module	X	X	X	X
Parent Quality of Life	X	X	X	X
Demographics	X			

All measures will be collected as outlined in Table 5. Our primary outcome measure is percent kilocalories obtained orally.

#### Percent kilocalories obtained orally

This measure will be obtained using the 24-h food recall taken over 3 days. The 24-h food recall is a standardized three-pass method, developed by the US Department of Agriculture for use in national dietary surveillance. As opposed to a food log or record (which is less scientifically rigorous), this recall is obtained over the phone by trained dietitians using specific probes to gather significant details about items consumed. Although there are weaknesses to every method of dietary assessment, this one was selected as it is widely used in several large trials and data suggest it is the most valid and reliable method of dietary assessment for children, and is considered the gold standard [21]. The data will be collected using standardized probes by trained research staff, and parents will be presented with paper food models and measuring devices prior to interviews to reference during the recall. Recalls will be rigorously analyzed with the Nutritional Data System for Research, version 2019; University of Minnesota, Minneapolis, MN. Although a plethora of information is available from this detailed, scientific analysis, the current study will focus on total daily oral calorie intake, and the percent of this intake consumed orally.

#### Morning cortisol

The blood for the morning cortisol test will be drawn before 8 am and run at the local site pediatric laboratory to test for adrenal insufficiency. Because each laboratory has its own reference range for morning cortisol depending upon the assay used, we will have labs report both the actual value in units and the category description (low/within normal limits/high).

#### Parent stress (The Pediatric Inventory for Parents—PIP [22])

Parent stress will be measured via the Pediatric Inventory for Parents, a 52-item parent questionnaire developed to measure parent stress around caring for a medically complicated child. The measure has a total and four domain scale scores (communication, medical care, role function, emotional function). For the purposes of the current study, we will assess the primary caregiver (the parent who does the majority of the care for the tube-fed child) and will use the total score, as well as the four domain scale scores. The measure has been shown to be a valid and reliable measure of pediatric illness-related parenting stress [22].

#### Impact of pediatric chronic health conditions (PedsQL 2.0, Family Impact Module)

The impact of pediatric chronic health conditions on parents and the family will be measured with the PedsQL 2.0, Family Impact Module (63) [23]. This 36-item survey measures parent self-reported physical, emotional, social, and cognitive functioning; communication; and worry. In addition, the Module assesses parent-reported family daily activities and family relationships.

#### Child quality of life (Infant Toddler Quality of Life—ITQOL [24])

Child quality of life will be measured with the Infant Toddler Quality of Life short-form questionnaire, validated for children 2 months through 5 years, 11 months, 31 days of age. The ITQOL has 47 items which result in 9 multi-item scales with well-established reliability (Cronbach’s alpha > .70) and validity. Similar to our previous work [16], we will use the multi-item scales in our analyses.

#### Parent quality of life (Parent Quality of Life SF-36v2)

Parent quality of life will be measured with the widely used Parent Quality of Life Short Form 36, version 2. Over 4000 studies have used the SF-36, making it a widely accepted measure [25]. The 36 questions with 5-point scales have 8 scaled scores: vitality, physical functioning, bodily pain, general health perceptions, physical role functioning, emotional role functioning, social role functioning, and mental health. As is typical, we will convert individual scores to *z*-scores, resulting in a standardized combined score [26]. This standardized combined score will be used in analyses, as well as specific subscales that previous research indicates may be sensitive to the current intervention (growth and development, behavior, general health perceptions, parental impact: emotion, parental impact: time).

#### Child health-related quality of life (Child Health Questionnaire Parent Form—CHQ-PF50)

Child quality of life in children ≥ 5 years old will be measured with the Child Health Questionnaire Parent Form questionnaire. The parent-reported CHQ is normed for ages 5 to 18 and measures 14 unique physical and psychosocial concepts. The CHQ has 50 items and scores for the parent-reported versions can be analyzed at the concept level (CHQ Profile Scores) or combined to derive an overall physical and psychosocial score (CHQ Summary Scores).

#### Demographics

Families will complete a demographic questionnaire regarding age, race, ethnicity, income, insurance status, and parental education, along with a medical history questionnaire asking about diagnoses at birth and diagnoses at week 0 only. These variables will be used to describe our sample and in any covariate analyses.

#### Post-treatment questionnaire

Therapies outside of the iKanEat protocol will be assessed via the post-treatment questionnaire at the final clinic visit (week 24) and be available for use in analyses as covariates if necessary.

#### Safety

To monitor for any negative side effects, patients will be contacted routinely (via the tele-visits and clinic visits described previously) for any negative gastrointestinal, behavioral, feeding, or other negative sequelae suspected to result from our treatment. Any negative outcomes identified will be reported to our Data Safety and Monitoring Board (DSMB; if required) and proper steps will be taken. Under certain conditions (10% body weight loss from the start of wean through any point later in the protocol, and/or irritability or behavioral change unacceptable to parent or evaluator), children will be removed from the study and treated clinically. At every clinic visit and tele-visit visit, we will record and evaluate concomitant medications. Should any clinical concerns be noted, appropriate medical/clinical treatments will also occur as necessary. If any serious adverse events occur, providers will be unblinded to the subject’s group, the subject will be removed from the study, and patients will receive all necessary treatment clinically.

#### Data Safety Monitoring Board

For the current study, we will have a Data Safety Monitoring Board that will review all subject safety data twice annually after the first 5 subjects are enrolled through the end of the study. Twice a year, the DSMB will review protocol adherence, adverse events, unanticipated problems, voluntary and study team initiated withdrawals, etc. This team is also available to the investigators at any time should an issue of safety arise. Any serious adverse events will be reported immediately to the relevant institutional IRBs and to the DSMB.

#### Unresolved adverse events

All unresolved adverse events should be followed by the investigator until the events are resolved, the subject is lost to follow-up, or the adverse event is otherwise explained. At the last scheduled visit, the investigator should instruct each subject to report any subsequent event(s) that the subject, or the subject’s personal physician, believes might reasonably be related to participation in this study. The investigator should notify the study sponsor of any death or adverse event occurring at any time after a subject has discontinued or terminated study participation that may reasonably be related to this study. The sponsor should also be notified if the investigator should become aware of the development of cancer or of a congenital anomaly in a subsequently conceived offspring of a subject that has participated in this study.

### Analysis plan

#### Sample size determination

Sample size determination was calculated using Power Analysis & Sample Size (PASS) software. If at least 90% of the treatment group successfully transitions (in pilot 100% successfully transitioned [16]), with 72 participants, we would have .80 power to detect an odds ratio of .21. With 60 participants, we would have power to detect an odds ratio of .18.

#### Planned analyses

Analyses will be conducted using Generalized Linear Mixed Models with a logit link within SAS Proc GLIMMIX to model the binary outcome of transitioned to oral feeding (y/n) defined as at least 90% of calories consumed orally. Fixed effects (dummy variables) will be entered for each site as described by McNeish and Stapleton to account for the clustering of the participants within sites [27]. This will result in a logistic regression model with appropriate standard errors. Sex, age at initiation, treatment condition, and interactions with treatment condition will be included in the models to enable us to examine the effect of group assignment on successful transition to oral feeding by week 24. Interactions between the treatment group and sex will be examined but will not be sufficiently powered to reach definitive conclusions about sex differences. In addition to overall effects, subgroup analyses will be reported for males and females to examine treatment effects within sex groups.

Should any participant have cortisol levels outside of the normal range, we will examine the individual characteristics associated with this, including group assignment, medical diagnoses, sex, and age at initiation. These characteristics will be reported descriptively.

We will model parent stress levels, child quality of life, and parent quality of life for participants in both treatment conditions over the four measurement occasions using General Linear Mixed Models. As in aim 1, fixed effects for the site will be added to the model as will sex and age at initiation. Once the shape of the trajectory over time has been modeled, a successful transition to oral feeding indicator, defined as consuming 90% or more of calories orally, will be added to the model at level 2. For 3A, the primary interest will be the time by successful transition interaction on parental stress level. Estimate statements will examine group differences in stress at weeks 14 and 24. To address hypothesis 3B regarding quality of life outcomes, we will initially model the trajectory over time for each outcome with the same set of predictors as in 3A.

#### Population(s) for analysis

Our primary analysis will focus on a protocol-compliant population, but we will secondarily also analyze the all-treated population, as described below.

- All-randomized population: Any subject randomized into the study, regardless of whether they received study drug
- All-treated population: Any subject randomized into the study that received at least one dose of study drug
- Protocol-compliant population: Any subject who was randomized and received the protocol required study drug exposure and required protocol processing

#### Data handling

Data will be collected using REDCap, which allows the ability to ensure responses have complete data. Study coordinators will verify the data during the clinic visit to ensure data collection was accurate and complete.

Site PI and site study coordinators will have access to their own site’s data. The lead PI and study team will have access to data from all sites. Sites will not have access to other site’s identified data. De-identified aggregate data may be shared for safety monitoring during site conference calls and to the DSMB. The lead Investigational Pharmacy will have access to identified data from each site for drug randomization.

Data will be shared from the sites to the coordinating site via REDCap or secure file transfer. Data will be saved onto secured shared drives at KUMC and on shared drives at the site’s home institutions. KUMC will retain the data for 10 years after the completion of the study or once subjects turn 18 years old.

Identifiable information will be collected. Information will be coded with a subject ID and identifiers will be removed to a linking list that will be saved in a separate location. The lead PI and study coordinator will have access to all coded data. Site PI and coordinators will have access to the linking log for their site only. Identified data will be located in REDCap. Once data are abstracted from REDCap, they will be saved on the KUMC shared drives where identifiers will be removed and placed into a linking log. Identifiable data will be sent from the site to KUMC using secure file transfer.

Data will be stored in REDCap and on the lead site secure shared drives. Consent will be obtained electronically via REDCap. REDCap may be brought up on a mobile device for participants to complete; however, no data will be stored directly on the mobile device.

#### Confidentiality

Information about study subjects will be kept confidential and managed according to the requirements of the Health Insurance Portability and Accountability Act of 1996 (HIPAA). Those regulations require a signed subject authorization informing the subject of the following:

- What protected health information (PHI) will be collected from subjects in this study
- Who will have access to that information and why
- Who will use or disclose that information
- The rights of a research subject to revoke their authorization for use of their PHI

In the event that a subject revokes authorization to collect or use PHI, the investigator, by regulation, retains the ability to use all information collected prior to the revocation of subject authorization. For subjects that have revoked authorization to collect or use PHI, attempts will be made to obtain permission to collect at least vital status (i.e., that the subject is alive) at the end of their scheduled study period.

### Ethics and dissemination

This study will be conducted according to US and international standards of Good Clinical Practice (FDA Title 21 part 312 and International Conference on Harmonization guidelines), applicable government regulations, and Institutional research policies and procedures. This protocol has been approved by the University of Kansas Medical Center’s Institutional Review Board (KUMC IRB), which is serving as the central IRB (through the SMART IRB system). Any protocol modifications are submitted to the KUMC IRB for approval and communicated to each of the participating sites. All subjects for this study will be provided a consent form describing this study and providing sufficient information for subjects to make an informed decision about their participation in this study. The formal consent of a subject, using the EC/IRB-approved consent form, must be obtained before that subject undergoes any study procedure. The consent form must be signed by the subject or legally acceptable surrogate, and the investigator-designated research professional obtaining the consent. The findings of the study will be presented at national conferences and published in peer-reviewed journals. The study sponsor and funder has no role in the collection, management, analysis, interpretation, or writing of the report. Professional writers will not be used in the composition of the manuscript.

## Discussion

Existing treatment options for children with feeding problems transitioning from tube to oral feeding are limited. Previous studies of our outpatient protocol (iKanEat) have demonstrated promising results, but the randomized controlled trial described herein is a critical next step in the assessment of the necessity of megestrol as a component of this treatment program. The results regarding the safety of megestrol will also likely be helpful to physicians and families considering the use of megestrol for other clinical populations, such as children with cystic fibrosis or those with cancer.

### Trial status

Actively recruiting

Protocol version and date: April 21, 2020

Date recruitment began: August 5, 2019

Approximate date recruitment will be completed: January 2023

## Data Availability

Data will be saved onto secured shared drives at the primary site. De-identified data will be available upon written request to the corresponding author.

## Abbreviations

BP: Blood pressure
DSMB: Data Safety Monitoring Board
etc.: Et cetera
FDA: Food and Drug Administration
G: Gastrostomy
GJ: Gastrojejunal
Ht: Height
ITQOL: Infant Toddler Quality of Life
KUMC: University of Kansas Medical Center
NASPGHAN: North American Society for Pediatric Gastroenterology, Hepatology & Nutrition
PI: Principle investigator
PIP: The Pediatric Inventory for Parents
POC: Point of contact
PHI: Protected health information
RedCap: Research Electronic Data Capture
SAE: Serious adverse event
SF: Short Form
Temp: Temperature
Wt: Weight

## References

[1] Rudolph CD, Link DT (2002). Feeding disorders in infants and children. Pediatr Clin N Am.

[2] Burklow KA, Phelps AN, Schultz JR, McConnell K, Rudolph C (1998). Classifying complex pediatric feeding disorders. J Pediatr Gastroenterol Nutr.

[3] Ishizaki A, Hironaka S, Tatsuno M, Mukai Y (2013). Characteristics of and weaning strategies in tube-dependent children. Pediatr Int.

[4] Lalanne A, Gottrand F, Salleron J, Puybasset-Jonquez AL, Guimber D, Turck D, Michaud L (2014). Long-term outcome of children receiving percutaneous endoscopic gastrostomy feeding. J Pediatr Gastroenterol Nutr.

[5] Pedersen S, Parsons H, Dewey D (2004). Stress levels experienced by the parents of enterally fed children. Child Care Health Dev.

[6] Avitsland TL, Faugli A, Pripp AH, Malt UF, Bjornland K, Emblem R (2012). Maternal psychological distress and parenting stress after gastrostomy placement in children. J Pediatr Gastroenterol Nutr.

[7] Silverman AH, Kirby M, Clifford LM, Fischer E, Berlin KS, Rudolph CD, Noel RJ (2013). Nutritional and psychosocial outcomes of gastrostomy tube-dependent children completing an intensive inpatient behavioral treatment program. J Pediatr Gastroenterol Nutr.

[8] Dunbar SB, Jarvis AH, Breyer M (1991). The transition from nonoral to oral feeding in children. Am J Occup Ther.

[9] Gutentag S, Hammer D (2000). Shaping oral feeding in a gastronomy tube-dependent child in natural settings. Behav Modif.

[10] RxMed: Megace: http://www.rxmed.com/b.main/b2.pharmaceutical/b2.prescribe.html. Accessed 2 Nov 2017.

[11] Marchand V, Baker SS, Stark TJ, Baker RD (2000). Randomized, double-blind, placebo-controlled pilot trial of megestrol acetate in malnourished children with cystic fibrosis. J Pediatr Gastroenterol Nutr.

[12] Edwards S, Davis AM, Ernst L, Sitzmann B, Bruce A, Keeler D, Almadhoun O, Mousa H, Hyman P (2015). Interdisciplinary strategies for treating oral aversions in children. JPEN J Parenter Enteral Nutr.

[13] Morris N, Knight RM, Bruni T, Sayers L, Drayton A (2017). Feeding disorders. Child Adolesc Psychiatr Clin.

[14] Byars KC, Burklow KA, Ferguson K, O'Flaherty T, Santoro K, Kaul A (2003). A multicomponent behavioral program for oral aversion in children dependent on gastrostomy feedings. J Pediatr Gastroenterol Nutr.

[15] Clawson EP, Kuchinski KS, Bach R (2007). Use of behavioral interventions and parent education to address feeding difficulties in young children with spastic diplegic cerebral palsy. NeuroRehabilitation.

[16] Davis AM, Dean K, Mousa H, Edwards S, Cocjin J, Almadhoun O, He J, Bruce A, Hyman PE (2016). A randomized controlled trial of an outpatient protocol for transitioning children from tube to oral feeding: no need for amitriptyline. J Pediatr.

[17] Cuvelier GD, Baker TJ, Peddie EF, Casey LM, Lambert PJ, Distefano DS, Wardle MG, Mychajlunow BA, Romanick MA, Dix DB, Wilson BA (2014). A randomized, double-blind, placebo-controlled clinical trial of megestrol acetate as an appetite stimulant in children with weight loss due to cancer and/or cancer therapy. Pediatr Blood Cancer.

[18] Couluris M, Mayer JL, Freyer DR, Sandler E, Xu P, Krischer JP (2008). The effect of cyproheptadine hydrochloride (periactin) and megestrol acetate (megace) on weight in children with cancer/treatment-related cachexia. J Pediatr Hematol Oncol.

[19] Orme LM, Bond JD, Humphrey MS, Zacharin MR, Downie PA, Jamsen KM, Mitchell SL, Robinson JM, Grapsas NA, Ashley DM (2003). Megestrol acetate in pediatric oncology patients may lead to severe, symptomatic adrenal suppression. Cancer.

[20] Eubanks V, Koppersmith N, Wooldridge N, Clancy JP, Lyrene R, Arani RB, Lee J, Moldawer L, Atchison J, Sorscher EJ, Makris CM (2002). Effects of megestrol acetate on weight gain, body composition, and pulmonary function in patients with cystic fibrosis. J Pediatr.

[21] Foster E, Bradley J (2018). Methodological considerations and future insights for 24-hour dietary recall assessment in children. Nutr Res.

[22] Streisand R, Braniecki S, Tercyak KP, Kazak AE (2001). Childhood illness-related parenting stress: the pediatric inventory for parents. J Pediatr Psychol.

[23] Varni JW, Sherman SA, Burwinkle TM, Dickinson PE, Dixon P (2004). The PedsQL™ family impact module: preliminary reliability and validity. Health Qual Life Outcomes.

[24] Landgraf JM, Vogel I, Oostenbrink R, van Baar ME, Raat H (2013). Parent-reported health outcomes in infants/toddlers: measurement properties and clinical validity of the ITQOL-SF47. Qual Life Res.

[25] Yamada A, Kato M, Suzuki M, Suzuki M, Watanabe N, Akechi T, Furukawa TA (2012). Quality of life of parents raising children with pervasive developmental disorders. BMC Psychiatry.

[26] Ware JE, Kosinski M, Bjorner JB, Turner-Bowker DM, Gandek B, Maruish ME. User’s manual for the SF-36v2 health survey. Lincoln: Quality Metric; 2008.

[27] Daniel M. McNeish, Laura M. Stapleton. The Effect of Small Sample Size on Two-Level Model Estimates: A Review and Illustration. Educ Psychol Rev. 2016;28(2):295–314.

